# Evaluation and Comparative Analysis of Coronal Tooth Discoloration Induced by Four Endodontic Biomaterials Utilized in Regenerative Endodontic Procedures: An Ex-vivo Study

**DOI:** 10.7759/cureus.62874

**Published:** 2024-06-21

**Authors:** Divya Mishra, Aishwarya Arya, Kanduri Venkata Naga Vamseekrishna, Bisma Jahangeer, Mayank Sachdeva, Aprajita Moses

**Affiliations:** 1 Dentistry, Heritage Hospitals, Varanasi, IND; 2 Conservative Dentistry and Endodontics, Maharishi Markandeshwar College of Dental Sciences and Research, Mullana, IND; 3 Conservative Dentistry and Endodontics, Awadh Dental College and Hospital, Jamshedpur, IND; 4 Conservative Dentistry and Endodontics, Care Dental College, Guntur, IND; 5 Conservative Dentistry and Endodontics, Private Practice, Kulgam, IND; 6 Conservative Dentistry and Endodontics, Ex-Servicemen Contributory Health Scheme (ECHS) Polyclinic, Kolkata, IND

**Keywords:** regenerative endodontic therapy, tooth discoloration, calcium hydroxide, mineral trioxide aggregrate, biodentin, intracanal coronal barrier

## Abstract

Aim: This study aims to compare and evaluate the changes in color caused by four different biomaterials utilized as intracanal coronal barriers in the regenerative endodontic procedure at varying intervals of time.

Methods: A total of 100 extracted mandibular single-rooted premolars were taken. Samples were standardized, and access cavity preparation was done. All the canals were instrumented and irrigated thoroughly. They were divided into five groups: A, B, C, D, and E (n=20), i.e., the control group, gray mineral trioxide aggregate (MTA), white MTA, Biodentine, and calcium hydroxide. Each group was further subdivided into two subgroups (n=10), where one subgroup was prepared with normal saline and the other was prepared with platelet-rich fibrin (PRF). Digital photographs were taken at baseline, immediately after the placement of biomaterials, after 72 hours, seven days, and six months, using a DSLR camera. The color evaluation was done using the Adobe Photoshop 2021 software using the CIE L*a*b color system. The data obtained were recorded and statistically analyzed using IBM SPSS Statistics for Windows, Version 23 (Released 2015; IBM Corp., Armonk, New York). Continuous variables were analyzed using a one-way ANOVA test and post-hoc analysis. The *P-*value was set to be significant at <0.05.

Results: Statistically significant variation was obtained in all four experimental groups regarding change in color (ΔE) over periods of 72 hours, seven days, and six months. At six months, all the experimental groups presented with a perceptible color variation (ΔE>3.3).

Conclusion: The color change was significant after six months in all groups. Biodentine produced the least color alteration, whereas gray MTA produced the highest.

## Introduction

The primary goal of treatment offered by a dentist is to restore optimal health, functionality, and aesthetics to the dentition. Currently, the management of pulpal necrosis in an immature tooth with an open apex presents one of the unique challenges to endodontists [1]. It is caused by trauma or carious exposure to the pulp, hindering the development process of the root and leading to the formation of shortened roots with thin walls. Eventually, this increases the tendency toward root fracture [2]. The ideal treatment in such cases would be regenerative endodontic procedures (REP) that can stimulate the regeneration of the pulp-dentin complex in an effort to obtain further development of the root in terms of thickness and length [3].

REP can be achieved through the activity of specific cells originating from dental pulp, stem cells, or the vascular and immune systems of the periodontium. These stem cells multiply in the root canal under favorable conditions, i.e., in the absence of microorganisms, their by-products, dead tissues, and the existence of a protein scaffold with a tight coronal seal. The most current treatment protocols involve the use of the host’s own pulp or vascular tissues [4].

In REP, the scaffold plays a pivotal role by selectively binding and localizing cells. Comprising growth factors, it undergoes biodegradation over time. Platelet-rich fibrin (PRF) has emerged as a successful physical scaffold in REP. Its high-density fibrin clot facilitates a gradual and sustained increase in cytokine levels, while the leukocytes within PRF serve as regulators of the immune response. Moreover, PRF serves as a source of vascular endothelial growth factor, stimulating angiogenesis [3].

Bioceramic materials are applied to establish a hard tissue barrier above the scaffold, isolating the root canal from the external tooth surface. Various calcium-silicate cements, such as mineral trioxide aggregate (MTA) and Biodentine, have been used as intracanal coronal barriers. They have shown outstanding biocompatibility, bioactivity, and good sealing ability [5].

Although REP is one of the best techniques, it has some ambiguities and limitations that need to be resolved. One of the drawbacks includes coronal tooth discoloration, likely induced by the placement of antibiotic paste, blood, or intracanal barrier material [6-8].

Therefore, the aim of this study is to compare and evaluate the change in color caused by four different biomaterials utilized as intracanal coronal barriers in REP, namely, calcium hydroxide, gray MTA, white MTA, and Biodentine at various time intervals.

## Materials and methods

A total of 100 intact human mandibular single-rooted premolar teeth, devoid of any carious lesions, previous restorations, extrinsic discolorations, cracks, or fractures, were collected for this study. The tooth samples underwent cleaning with an ultrasonic scaler (Satelec, Chennai, India) to eliminate calculus, soft tissue remnants, extrinsic stains, and debris, followed by polishing with pumice paste (DPI, Mumbai, India) and water. Subsequently, all samples were stored in a 0.9% saline solution (Lifusion, India) until use.

Horizontal resection of the root end in all tooth specimens was performed using a diamond disc (Contiene, India) from the buccal cementoenamel junction (CEJ) to achieve a standardized length of 10 mm. Access cavity preparation was carried out with a round bur (Mani, Tochigi, Japan) under high-speed conditions and with a water coolant. Thorough instrumentation, enlargement, and standardization of all canals were then performed using Gates Glidden drill #4 (Mani, Tochigi, Japan) along the entire crown-to-root length. Following this, the cavities were irrigated with 5 mL of 3% NaOCl (Prime, Chennai, India), followed by 5 mL of 17% EDTA (DPI, Mumbai, India), and finally rinsed with 5 mL of saline solution. Sterile paper points (Dentsply Sirona, Fair Lawn, New Jersey) were utilized for root canal drying. Aseptic cotton pellet placement within the access cavity extended to the CEJ, which was then sealed with provisional restoration (Cavit G, 3M ESPE, Irvine, California). The specimens were subsequently stored in glass bottles containing saline solution until further use.

The specimens were then randomly assigned to four experimental groups and a control group (A, B, C, D, and E; n=20). Further stratification occurred with each group subdivided into two subgroups (n=10), denoted as a1, a2, b1, b2, c1, c2, d1, d2, e1, and e2, employing a stratified random sampling approach. Following reassessment, removal of cotton pellets, and repetition of the irrigation regimen, the canals were thoroughly dried using paper points.

Preparation of subgroups with PRF

A total of 10 mL of whole blood was voluntarily collected from a random patient after obtaining consent from specialist medical personnel through venipuncture in sterile blood collection tubes. The whole blood was immediately centrifuged (REMI Medico Plus, Mumbai, India) at 2700 rpm for 13 minutes to separate the PRF. The PRF was then collected and injected into the canal space using an insulin syringe (Nipro, Osaka, Japan) directly at a level approximately 4 mm below the buccal CEJ in the experimental groups. A sterile collagen plug (ColoGenesis, Salem, India) was cut to a length of 2 mm and placed as a matrix over the PRF. The test materials were mixed according to the manufacturer's guidelines and placed into the cavities with a thickness of 3 mm, covering the collagen plug and extending up to the labial CEJ.

Preparation of subgroups with saline

A saline solution was used to fill the root canals, followed by the precise placement of a sterile collagen plug positioned snugly 3 mm below the CEJ. Subsequently, the test materials were carefully placed over the collagen plug.

Preparation of the control group

A total of 20 teeth were used as the control group wherein PRF for subgroup a1 (n=10) and saline for subgroup a2 (n=10) were placed in the canal space up to the CEJ and sealed with the same temporary restorative material. The samples were stored under conditions of 37 °C temperature and 100% humidity.

**Figure 1:**
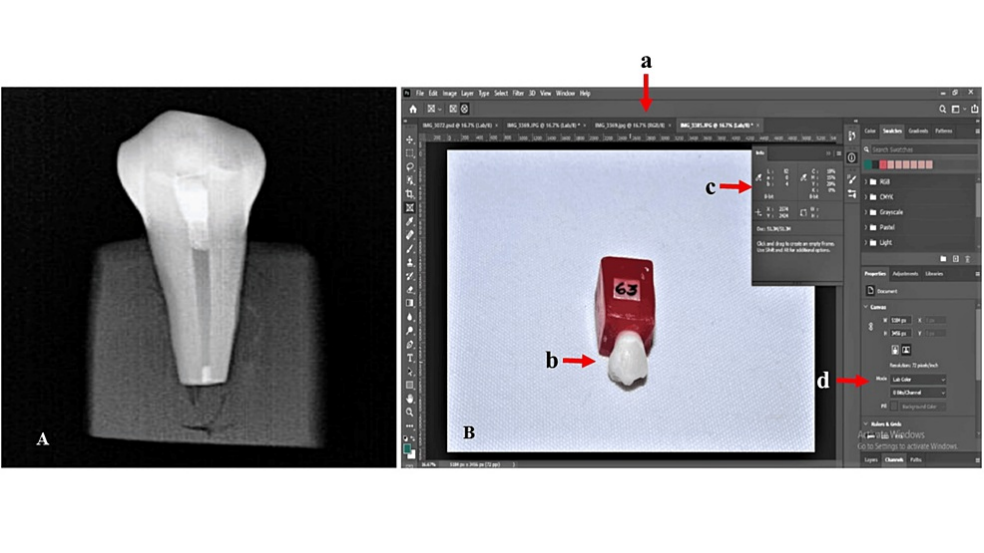
(A) Placement of experimental materials; (B) measurement of L*a*b coordinates of the CIELAB color a: Adobe Photoshop Software 2021 System; b: uploaded photograph model in the software for analysis; c: panel to display L*a*b coordinates value; d: selection panel of L*a*b coordinates system

Photographs were taken using a Canon EOS 1300D DSLR camera set at F22 aperture, 1/25 shutter speed, ISO 100, and flash at manual ½ power in a semi-dark room with a white background, positioning the emitter at an angle of 45°. The central circular area of the buccal surface of the samples was considered for photographic records. The color change was evaluated at different time intervals: T0 (baseline), T1 (immediately after the placement of biomaterial), T2 (72 hours later), T3 (seven days later), and T4 (six months later). Adobe Photoshop Software 2021 System was utilized for color analysis, following the protocols of the Commission Internationale de l’Eclairage (CIE) L*a*b* system. Color variation between the baseline and subsequent periods was quantified using the formula: ΔE = ((ΔL)² + (Δa)² + (Δb)²)1/2 (Figure 1).

Statistical analysis

Descriptive statistics were compiled. The data were analyzed using IBM SPSS Statistics for Windows, Version 23 (Released 2015; IBM Corp., Armonk, New York). Continuous variables were compared using a one-way ANOVA test and frequency distribution. Significance was defined by p-values less than 0.05 using a one-way ANOVA and post-hoc analysis.

## Results

Table 1 presents the results of the study conducted on various experimental subgroups over four different time points (T1, T2, T3, T4) in REP. Each subgroup represents a specific combination of materials used for intracanal procedures, including saline, PRF, gray MTA, white MTA, Biodentine, and calcium hydroxide. The mean color change (± standard deviation) at each time point is provided, along with the corresponding p-values indicating the statistical significance of the observed changes. Notably, significant color changes were observed in several subgroups, particularly at T2, T3, and T4, indicating a time-dependent effect on discoloration. For instance, PRF (subgroup a2) exhibited a statistically significant increase in color change compared to saline (subgroup a1) at all time points, highlighting its potential role in discoloration over time (p<0.05). Similarly, subgroups involving gray MTA (b1, b2) and white MTA (c1, c2) displayed significant color changes, with gray MTA demonstrating the most significant overall color change (p<0.05). Contrastingly, subgroups involving Biodentine (d1, d2) and calcium hydroxide (e1, e2) exhibited comparatively lower color changes, particularly at later time points, suggesting their potential for reduced discoloration (p<0.05) (Table 1).

**Table 1:** Mean and standard deviation of ΔE values across all groups throughout the evaluation periods *p<0.05 considered significant

Subgroups	T1	p-value	T2	p-value	T3	p-value	T4	p-value
a1 saline	2.7114±1.02645	0.052	3.2143±1.78512	0.051	3.1294±1.10910	0.054	3.1165±1.04734	0.571
a2 PRF	4.8885±0.94419	0.055	6.0927±1.28184	0.003*	6.7613±1.32710	0.036*	6.8463±0.95896	0.041*
b1 saline/gray MTA	4.7066±2.87674	0.056	5.7397±4.44173	0.028*	7.1837±4.53319	0.044*	7.2649±4.29753	0.022*
b2 PRF/gray MTA	4.8312±1.71899	0.065	8.3594±4.29741	0.047*	8.5052±4.89460	0.038*	9.1529±4.69339	0.036*
c1 saline/white MTA	2.6632±1.29422	0.054	4.9142±3.04599	0.021*	5.0979±1.91805	0.033*	5.5532±2.15045	0.012*
c2 PRF/white MTA	2.6632±2.60660	0.051	7.1237±2.77954	0.066*	8.4924±3.88441	0.037*	8.9164±2.53793	0.001*
d1 saline/Biodentine	2.5848±1.53434	0.053	3.1377±2.61514	0.048*	4.7262±2.53058	0.025*	4.9726±2.99512	0.033*
d2 PRF/Biodentine	4.1839±1.76213	0.057	4.9223±1.58833	0.038*	6.2174±2.65502	0.048*	6.3263±1.72512	0.043*
e1 saline/calcium hydroxide	2.6109±1.72671	0.058	4.2491±1.43186	0.027*	4.8209±1.78238	0.045*	5.0065±2.19468	0.001*
e2 PRF/calcium hydroxide	3.4105±1.81622	0.056	4.9667±2.14265	0.027*	6.6962±3.40933	0.038*	6.7701±2.57464	0.001*

**Figure 2:**
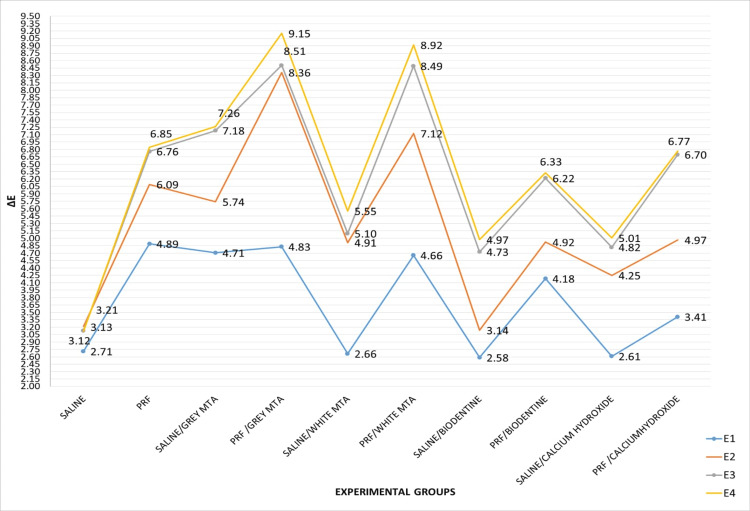
Relative variation of Commission International de l’Éclairage (CIE) parameter ΔE value of all groups measured at T1 (E1), T2 (E2), T3 (E3), and T4 (E4)

Figure 2 illustrates the relative variation of the Commission Internationale de l'Éclairage (CIE) parameter ΔE value across all groups at different time points: T1 (E1), T2 (E2), T3 (E3), and T4 (E4). The statistical analysis revealed significant variation in ΔE values across all four experimental groups over the 72-hour, seven-day, and six-month time periods, with ΔE values generally increasing over time. By the six-month mark, all experimental groups exhibited noticeable color variations (ΔE>3.3). Following the six-month evaluation, the gray MTA/PRF group (subgroup b2) displayed the highest ΔE value (9.15) among all groups. In the experimental groups treated with saline, Biodentine/saline demonstrated the lowest ΔE value (4.9), while the gray MTA/saline exhibited the highest ΔE value (7.26) at the six-month mark. Among the experimental groups treated with PRF, the gray MTA/PRF showed the highest ΔE value (9.15), while Biodentine/PRF displayed the lowest ΔE value (6.32) (Figure 2).

## Discussion

In the current landscape, regenerative endodontics has emerged as a cornerstone in the management of immature permanent teeth presenting with apical periodontitis and sinus tract complications [2]. This approach orchestrates the intricate interplay of growth factors, stem cells, and scaffolds to facilitate the restoration of tissue integrity [3]. A distinctive advantage inherent to regenerative endodontics lies in its streamlined methodology, leveraging readily available resources and medicaments for implementation.

Coronal tooth discoloration associated with REP has been widely recognized in previous studies [1,6,9]. The literature provides a good description of the relationship between materials used as intracanal coronal barriers and changes in hue over time [10]. Hence, the aim was to evaluate the color stability of four biomaterials that can be used as an intracanal coronal barrier in REP over a 6-month period.

PRF was the physical scaffold used in our study. According to Tsay et al., a slow and steady rise in cytokine levels is linked to this high-density fibrin clot [11]. According to Thibodeau et al., leukocytes in PRF regulate the immunological response and serve as a source of vascular endothelial growth factor (VEGF), a growth factor that aids in angiogenesis [12].

In the study, four different bioactive materials (gray MTA, white MTA, Biodentine, and calcium hydroxide) were used as intracanal coronal barriers. The location of these materials was kept at 3-4 mm apical to CEJ in support of the study conducted by Banchs and Trope in 2004 [4].

MTA is a bioactive silicate with hydrophilic particles. Its main constituents include dicalcium silicate-tricalcium aluminate, tricalcium silicate, tricalcium oxide, bismuth, and iron compounds. MTA has better sealing abilities [13], is biocompatible, and may also supply signaling molecules for the development of stem cells [6]. It is associated with conductive and inductive properties. Previous studies have also used MTA as an intracanal coronal barrier in REP to analyze its discoloration potential [14-16]. Biodentine, a bioceramic material with easy handling characteristics that induces odontoblastic differentiation and stem cell proliferation, was used in the study. Previous studies have also studied Biodentine for its staining potential, particularly in REP [14-17]. Another material used was calcium hydroxide, chosen for its biocompatibility and increased release of growth factors from dentin. Lenherr et al., in their study, compared the discoloration potential of various endodontic materials and inferred that calcium hydroxide has less staining potential [6].

Color assessment was performed using the digital photographic method. Digital photographs were taken using a DSLR camera, hailed as the best camera option for dental photography in previous studies [15,16]. Researchers have employed DSLRs with telephoto lenses of 100 mm macro settings because they consistently produce results that adhere to the "through the lens" theory. A DSLR also enables the customization of various lenses according to the situation. According to Miyajiwala et al., in a clinical setting, digital photography could replace spectrophotometers as a practical shade-selection tool [18].

The CIELAB color space was used to analyze the color change using the Adobe Photoshop 2021 software. It was designed as a space where a given numerical change correlates to a comparable perceptual change in color. It is acknowledged as an ISO standard for assessing and quantifying color, vision, and light [16].

The results did not show statistically significant changes (p<0.05) in ΔE values for all the experimental groups immediately after the placement of biomaterials (T1). Similar findings were reported in studies conducted by Chen et al. (2020) [15] and Palma et al. (2020) [16]. Statistically significant ΔE values were obtained for all the experimental groups at 72 hours (T2) and seven days (T3). Greater ΔE values were obtained with the gray MTA/PRF group and least with the Biodentine/saline group. The observed statistically significant color changes may stem from the presence of bismuth within the material composition, which undergoes reduction from its oxide state to a metallic form, culminating in the formation of a black compound and consequent tooth discoloration. Alternatively, exposure to a potent oxidizing agent could prompt bismuth to oxidize, yielding bismuth carbonate, which, upon light exposure, precipitates as a black substance. The superior color stability exhibited by Biodentine may be attributed to the incorporation of zirconium oxide as a radiopacifier rather than bismuth oxide [16]. Previous studies by Palma et al. in 2019 [19] and Palma et al. in 2020 [16] have also shown results similar to our studies.

At six months (T4), ΔE values were statistically significant for all the experimental groups. Greater ΔE values were obtained with the gray MTA/PRF group and least with the Biodentine/saline group. The potential elucidations, in conjunction with those previously mentioned for statistically significant color alterations, may revolve around the prolonged setting duration inherent to MTA. This extended setting period might facilitate erythrocyte adsorption and subsequent hemolysis, culminating in both tooth and material discoloration. Conversely, Biodentine boasts a shorter setting time of approximately 12 minutes, potentially curtailing blood absorption. This expedited setting of the hydraulic substance could account for the heightened color stability observed in the Biodentine cohort compared to the MTA group. Furthermore, there is speculation that the iron concentration of white MTA could oxidize into a calcium alumino-ferrite phase, which could induce a color shift on integration with dentinal tubules [20].

ΔE has changed significantly (p<0.05) for all the experimental groups in an increasing pattern individually. Biodentine showed the least color alteration, followed by calcium hydroxide, white MTA, and gray MTA. The PRF group showed more discoloration than the saline group over time. Similar results were obtained in some of the previous studies [15,16,19,21-23]. The potential rationales lie in the biomaterial's constitution, particularly the presence of bismuth oxide, known to trigger tooth discoloration upon interaction with potent oxidizing agents such as dentin collagen and sodium hypochlorite. Indeed, prior investigations consistently underscore MTA's inferior color stability relative to alternative calcium-silicate-based cement [16].

A possible colorant responsible for the observed change in color is PRF. The hemolysis of erythrocytes that penetrated the dentin and caused the deposition of hematin molecules in the dentinal tubules is thought to be the cause of the discoloration. Teeth stained with erythrocytes exhibit a color gradient, as evidenced by histochemical testing, with the most intense discoloration near the pulp chamber and decreasing intensity moving outward [16]. The heightened discoloration attributed to PRF exposure may be linked to the presence or absence of the smear layer and the material's porosity. A smear layer can either increase or decrease dentin permeability, thereby influencing discoloration intensity [15].

Given the imminent possibility of PRF exposure during REP, it is imperative to elucidate its potential interplay with these materials. Such insights are pivotal for precise biomaterial selection tailored to individual clinical scenarios, thereby ensuring superior treatment outcomes. In essence, the meticulous choice of biomaterial is pivotal in achieving optimal aesthetic results.

Observations spanning six months reveal Biodentine's minimal propensity for discoloration, contrasting with gray MTA's compromised color stability. Furthermore, previous research indicated Biodentine's potential for immediate bonding procedures owing to its abbreviated setting time (12 minutes post-placement), potentially augmenting procedural longevity.

In light of our ex-vivo investigation, it is evident that Biodentine stands out as the superior intracanal coronal barrier in REP, offering minimal discoloration and improved aesthetic outcomes. Despite our rigorous efforts, this study is not without limitations. As it was conducted ex vivo, it failed to replicate the dynamic oral environment. Ex-vivo models lack positive pulp pressure, potentially restricting blood flow to peripheral areas. Therefore, it is crucial to assess the impact of blood exposure under in-vivo conditions.

Additionally, a larger sample size and an extended evaluation period could have enhanced the study's robustness. The use of artificial saliva instead of normal saline might have better simulated oral conditions. Furthermore, the absence of post-test content analysis and the limited scope of color evaluation methods suggest areas for improvement. Unforeseeable factors may have influenced the study despite our meticulous efforts to ensure precision. Hence, further research is warranted to corroborate the conclusions drawn from this study.

## Conclusions

Within the constraints of our study, the results point to blood exposure as a potential contributing factor to the color variation of biomaterials as well as the composition of the material itself, following a six-month evaluation period. The material least likely to discolor over time was Biodentine. Gray MTA exhibited the most significant color change, subsequently causing a noticeable worsening of tooth discoloration over time. The selection of intracanal cervical barrier material used in REP must take into account not only the clinical and radiological outcomes but also the aesthetic consequences. Uncertainties arise in this instance due to the potential for blood contact, unexpected staining of the cervical barrier, and, subsequently, of the dental framework. In REP, Biodentine is a potentially effective alternative to MTA for use as a cervical barrier material. Further research is required to validate these findings in the current clinical scenario.
